# Biogeographic Distribution Patterns of the Archaeal Communities Across the Black Soil Zone of Northeast China

**DOI:** 10.3389/fmicb.2019.00023

**Published:** 2019-01-25

**Authors:** Junjie Liu, Zhenhua Yu, Qin Yao, Yueyu Sui, Yu Shi, Haiyan Chu, Caixian Tang, Ashley E. Franks, Jian Jin, Xiaobing Liu, Guanghua Wang

**Affiliations:** ^1^Key Laboratory of Mollisols Agroecology, Northeast Institute of Geography and Agroecology, Chinese Academy of Sciences, Harbin, China; ^2^State Key Laboratory of Soil and Sustainable Agriculture, Institute of Soil Science, Chinese Academy of Sciences, Nanjing, China; ^3^Department of Animal, Plant and Soil Sciences, AgriBio Centre for AgriBiosciences, La Trobe University, Bundoora, VIC, Australia; ^4^Department of Physiology, Anatomy and Microbiology, La Trobe University, Melbourne, VIC, Australia; ^5^Research Centre for Future Landscapes, La Trobe University, Melbourne, VIC, Australia

**Keywords:** 16S rRNA gene, Illumina MiSeq sequencing, Mollisols, real-time PCR, *Thaumarchaeota*

## Abstract

Although archaea are ubiquitous in various environments, the knowledge gaps still exist regarding the biogeographical distribution of archaeal communities at regional scales in agricultural soils compared with bacteria and fungi. To provide a broader biogeographical context of archaeal diversity, this study quantified the abundance and community composition of archaea across the black soil zone in northeast China using real-time PCR and high-throughput sequencing (HTS) methods. Archaeal abundances across all soil samples ranged from 4.04 × 10^7^ to 26.18 × 10^7^ 16S rRNA gene copies per gram of dry soil. Several soil factors were positively correlated with the abundances including soil pH, concentrations of total C, N, and P, and available K in soil, and soil water content. Approximately 94.2, 5.7, and 0.3% of archaeal sequences, and 31, 151, and 3 OTUs aligned within the phyla *Thaumarchaeota, Euryarchaeota*, and *Crenarchaeota*, respectively. Within the phylum of *Thaumarchaeota*, group 1.1b was a dominating genus accounting for an average of 87% archaeal sequences and phylogenetically classified as *Nitrososphaera*, a genus of ammonia oxidizing archaea. The response of dominating OTUs to environmental factors differed greatly, suggesting the physiological characteristics of different archaeal members is diversified in the black soils. Although the number of OTUs was not related with any particular soil parameters, the number of OTUs within *Thaumarchaeota* and *Euryarchaeota* was marginally related with soil pH. Archaeal community compositions differed between samples, and a Canonical correspondence analysis (CCA) analysis indicated that soil pH and the latitude of sampling locations were two dominating factors in shifting community structures. A variance partitioning analysis (VPA) analysis showed that the selected soil parameters (32%) were the largest drivers of community variation, in particular soil pH (21%), followed by geographic distances (19%). These findings suggest that archaeal communities have distinct biogeographic distribution pattern in the black soil zone and soil pH was the key edaphic factor in structuring the community compositions.

## Introduction

Archaea is the third domain of life containing unique characteristics not found in bacteria and eukarya (Woese et al., 1990). Previously, archaea were considered to dominate in extreme environments (Woese et al., 1990; DeLong, 1998), but the development of molecular techniques has led to the realization that archaea are ubiquitous (Ochsenreiter et al., 2003; Oline et al., 2006; Auguet and Casamayor, 2008; Youssef et al., 2012). Archaeal communities differ between environments. For example, marine sediments are dominated by the uncultured miscellaneous crenarchaeotal group (MCG) and the marine benthic group-D (MBG-D) (Lloyd et al., 2013), while methanogenic *Euryarchaeota* are the most dominant archaeal phyla in lake or wetland sediments (Li et al., 2017; Zhang et al., 2018). Upland soils, in contrast, are dominated by the newly named phylum *Thaumarchaeota* (formerly described as mesophilic *Crenarchaeota*) (Brochier-Armanet et al., 2008), which contains all known ammonia-oxidizing archaea (AOA) (Pester et al., 2011; Stahl and de la Torre, 2012).

Archaeal communities are ubiquitous in soils, and play key roles in the global geochemical cycles and affect greenhouse gas emissions. It has been shown that methanogenesis and anaerobic methane oxidation are specifically performed by anaerobic archaea in the carbon cycle, while in the nitrogen cycle the oxidation of ammonia to nitrite is conducted by *Thaumarchaeota* (Offre et al., 2013). However, the information about the compositions and distribution patterns of archaeal communities in agriculture soils is still in its infancy compared to that of bacterial and fungal communities. Some local, regional, and global scale surveys have shown that archaeal communities, including AOA communities, are driven by various environmental factors, such as pH (Nicol et al., 2008; Tripathi et al., 2013), salinity (Auguet et al., 2010), the C/N ratio (Bates et al., 2011), climate and vegetation cover (Angel et al., 2010), elevation (Singh et al., 2012), and multiple factors (Zheng et al., 2013). However, most of these studies were conducted using low-resolution molecular fingerprinting methods, which lack the broad coverage and in-depth data analysis compared to the HTS method used to study bacterial and fungal communities. Recently, the number of studies on the biogeographic distribution of the archaeal communities in soils has increased with the development of archaea-specific primer sets (Singh et al., 2012; Wang et al., 2015; Shi et al., 2016; Siles and Margesin, 2016; Ma et al., 2017). It should be noted that, while most of the studies focused on soil samples collected from non-disturbed natural environments, studies using the HTS method to reveal the biogeographic distribution patterns of archaea in agricultural soils are limited (Tripathi et al., 2013; Zhang et al., 2018).

Black soils, classified as Chernozems or Mollisols based on the WRB soil classification system, are highly fertile and agriculturally productive soil resources in China (Liu et al., 2012). The black soils are primarily distributed in Heilongjiang, Jilin and Liaoning Provinces of northeast China. This long and narrow area is called the black soil zone and covers ~900 km from the north to south and 300 km from the east to the west. Across the black soil zone, the annual average temperature decreases and the soil chemical fertility generally increases latitudinally from south to north (Zhang et al., 2007), which creates an ideal region to study the biogeographic distribution of soil microorganisms.

Our previous studies revealed the biogeographic distribution patterns of the bacterial and fungal communities (Liu et al., 2014, 2015), as well as the ammonia-oxidizing bacteria (AOB) and archaea (AOA) across the black soil zone in northeast China (Liu et al., 2018). We found that both the bacterial and fungal communities showed distinct geographic distribution in this region, and the soil pH and carbon content are two overarching edaphic factors that determine the distribution of the bacterial and fungal communities, respectively (Liu et al., 2014, 2015). For ammonia oxidizers, we detected that the AOA showed more distinct biogeographic distribution patterns than the AOB across the black soil zone, and the soil pH was the predominant soil factor in shaping both the AOA and AOB community structures (Liu et al., 2018). However, the compositional and biogeographic distribution patterns of the archaea in this region have not been addressed.

In this study, the same soil samples from our previous studies on the bacterial and fungal communities were used to analyze the abundance and community compositions of the archaea across the black soil zone using real-time qPCR and Illumina MiSeq sequencing methods. The objectives of this study were (1) to reveal the biogeographic distribution pattern of the archaeal communities across the black soil zone; (2) to examine the environmental factors that shaped the distribution of the archaeal communities, and (3) to compare the differences and similarities of the archaeal communities across the black soil zone.

## Materials and Methods

### Soil Sampling, Physicochemical Property, and DNA Extraction

Soil sampling and the determination of soil physicochemical properties were conducted as previously described (Liu et al., 2014). In brief, based on the China Black Soil Ecology database (http://www.blackland.csdb.cn), 26 arable soil samples were collected across the black soil zone of northeast China in September 2012 (Figure S1). Each soil sample was a composite of 10 soil cores that were randomly collected from tillage layers (0–20 cm) within an area of ~100 m^2^. DNA was extracted from 0.5 g moist soil using an E.Z.N.A Soil DNA kit (OMEGA, USA) according to the manufacturer's instructions. The extracted DNA was diluted in TE buffer (10 mM Tris-HCl, 1 mM EDTA, adjust pH to 7.0) and stored at−20°C until used for additional analyses. Soil physicochemical properties are listed in Table S1.

### Quantitative PCR Analysis

Archaeal abundance was quantified using real-time PCR targeting the hypervariable V3–V4 regions of the 16S rRNA gene with the primers Arch519f (5′-CAG CCG CCG CGG TAA-3′) (Øvreås et al., 1997) and Arch915r (5′-GTG CTC CCC CGC CAA TTC CT-3′) (Stahl and Amann, 1991; Inceoglu et al., 2015). Each PCR reaction contained 10 μL of SYBR Premix Ex Taq^TM^ (Takara, Dalian, China), 0.4 μL of 10 μM each forward and reverse primers, 2 μL of extracted soil DNA, and the volume was reached to 20 μL with sterilized MilliQ water. Amplification was performed in a LightCycler^®^480 (Roche Applied Science) using initial denaturation at 95°C for 30 s, followed by 30 amplification cycles (95°C for 30 s, 60°C for 40 s, 70°C for 30 s), and a final extension at 50°C for 5 min for cooling. Standard curves were generated using a 10-fold dilution series of known concentrations using a plasmid containing the archaeal 16S rRNA gene amplicon. The copy number of the archaeal genes was calculated by a regression equation to convert the cycle threshold value to the known copy numbers in the standard curves. The copy numbers of the archaeal genes were converted into per gram of dry soil as described in our previous study (Liu et al., 2018). In addition, the ratio of between the archaeal and bacterial 16S rRNA gene copies was also calculated using abundance data of bacterial 16S rRNA genes reported previously (Liu et al., 2016a).

### Illumina MiSeq Sequencing

Archaeal 16S rRNA genes were PCR amplified using the primer pairs Arch519f/Arch915r (Bai et al., 2017), with the forward primer being modified to contain a unique 8 nt barcode at the 5′ end. PCR reactions were performed in triplicate in a 25 μL mixture containing 1.0 μL purified DNA template (1–10 ng), 0.5 μL of each primer (10 μM), and 23 μL of Platinum PCR SuperMix (TransGen Biotech Co. Ltd., Beijing, China). Each sample was amplified under the following conditions: initial denaturation at 95°C for 5 min, followed by 35 cycles (95°C for 1 min, 60°C for 45 s, 70°C for 1 min) with a final extension at 72°C for 5 min. Each PCR product was pooled and purified using an Agarose Gel DNA purification kit (TaKaRa, Dalian, China). Equimolar amount of the PCR products were combined into one pooled sample and prepared for sequencing (2 × 300) using an Illumina MiSeq platform at Majorbio BioPharm Technology Co., Ltd. (Shanghai, China).

### Bioinformatics Analysis

The raw data were processed and analyzed using QIIME Pipeline (http://qiime.org/tutorials/tutorial.html) (Caporaso et al., 2010b). Original paired reads were joined with FLASH (fast length adjustment of short reads) software (Magoč and Salzberg, 2011). Low quality sequences which shorter than 200 bp, and with an average base quality score of <20 were removed for further analysis. The trimmed sequences were chimera-detected and removed with the flags - -non_chimeras_rentention = intersection basing on a combination of *de novo* and reference-based chimera checking. The high-quality sequences were clustered into OTUs with UCLUST based on a 97% similarity level using QIIME's pick_ open_reference_otus.py (Edgar et al., 2011). The taxonomic classification of each phylotype (OTU) was aligned using the Python Nearest Alignment Space Termination (PyNAST) tool (Caporaso et al., 2010a) and a relaxed neighbor-joining tree built by FastTree (Price et al., 2009). The taxonomic identity of each phylotype was determined using the Ribosomal Database Project (RDP) classifier with a confidence threshold of 0.80 (http://rdp.cme.msu.edu/) (Cole et al., 2005). After the taxonomies had been assigned, the OTUs not matched to the archaea were removed from the dataset. The raw reads obtained in this study were submitted to the National Center for Biotechnology Information (NCBI) Sequence Read Archive (SRA) with accession number SRP139400.

### Statistical Analysis

The OTU richness was used to compare soil archaeal alpha diversity. The SPSS software (version 18.0 for Windows) was adopted to conduct Pearson's correlation analysis to identify possible correlations between abundance of archaeal genes, alpha diversity, relative abundance of the taxonomic subgroups (relative abundance >0.3% at least in one sample) and soil characteristics. In order to clarify the taxonomic status of *Thaumarchaeota* and archaea, the phylogenetic identities of each phylotype obtained in this study were aligned with the taxonomy-determined sequences obtained from the NCBI database using ClustalX 1.81 (Thompson et al., 1997). Briefly, the alignment analysis was performed with ClustalX 1.81, and a rooted neighbor-joining tree of *Thaumarchaeota* and an unrooted phylogenetic tree of archaea was constructed using Molecular Evolutionary Genetic Analysis software (MEGA 7.0) with 1,000-fold bootstrap support (Kumar et al., 2016). Meanwhile, an unrooted phylogenetic tree was illustrated by the interactive Tree of Life online program (Letunic and Bork, 2006).

To correct for sampling effort, we used a randomly selected subset of 8,300 archaeal sequences per sample to calculate the archaeal community compositions. Non-metric multidimensional scaling (NMDS) was used to analyze beta diversity in different soil samples, which was conducted on the basis of the calculated weighted UniFrac distances (Lozupone and Knight, 2005). Canonical correspondence analysis (CCA) was used to test the significant effects of environmental variables that drive the difference in the archaeal communities with latitude, soil water content, total C, N, and P, C/N, pH, available K and P, NH${}_{4}^{+}$-N, and $\text{N}{\text{O}}_{3}^{-}$-N as the explanatory factors. Only soil factors which had significant effects on the archaeal communities were retained for the CCA analysis that was selected using the *bioenv ()* function in the R environment. In addition, a VPA was conducted to quantify the relative contributions of the special factors and environmental variables on the archaeal community compositions using the “vegan” package in R 2.3.5 (R Development Core Team, 2010).

## Results

### Abundance of Archaea Across Black Soils

The archaeal abundance of 16S rRNA gene in all soil samples ranged from 4.04 × 10^7^ to 26.18 × 10^7^ copies per gram of dry soil (Table S1). The ratio between the archaeal and bacterial 16S rRNA gene copies ranged from 0.032 to 0.085. Pairwise correlation analysis shown that the abundance of archaea highly correlated with soil pH (*r* = 0.516, *p* = 0.007), soil C content (*r* = 0.821, *p* < 0.0001), soil N content (*r* = 0.757, *p* < 0.0001), soil water content (*r* = 0.676, *p* < 0.0001), available K content (*r* = 0.609, *p* = 0.001), and soil P content (*r* = 0.629, *p* = 0.001) (Figure S2).

### Taxonomic Classification of Archaea

In total, 735,020 high-quality archaeal sequences were obtained from all 26 samples. Of these, 74.12% could be classified as archaeal sequences by classifier alignment using the RDP database, and 8,313–38,342 sequences were obtained per sample (mean 25,885). The read lengths ranged from 381 to 419 bp with a mean of 384 bp.

*Thaumarchaeota* was the dominant archaeal phylum accounting for an average of 94.15% of the total observed sequences (ranging from 85.44 to 98.86%). *Euryarchaeota* was the second most abundant phylum with an average of 5.72% across all the samples (ranging from 1.14 to 13.29%). *Crenarchaeota* was sporadically detected in 10 soil samples with a relative abundance below 0.31%, with the exception of a location of LS with a relative abundance of 2.74% (Table S2). None of the other archaeal phyla were detected in the black soils.

Within the phylum of *Crenarchaeota*, only two orders *Desulfurococcales* and *Thermoproteales* were occasionally observed in the black soils (Table S2). Within the *Euryarchaeota*, 10 orders were observed with *Methanomassiliicoccales* detected in all soil samples with a relative abundance ranging from 0.75 to 5.87% (average of 3.03%), followed by *Methanobacteriales* ranging from 0.24 to 5.50% (average of 2.04%). Other orders were sporadically detected in some soil samples with very low abundances. Within *Thaumarchaeota*, three orders were detected, of which *Nitrososphaerales* was the most abundant with a relative abundance that ranged from 58.52 to 97.55% (average abundance of 87.35%), followed by *Thaumarchaeota*_norank with an average of 6.27%; *Nitrosopumilales* was only detected in 7 of the 26 samples with a relative abundance below 0.6%, with the exception of two samples of CT1 and LS with a relative abundance of 5.41 and 7.81%, respectively (Table S2).

**Figure 1:**
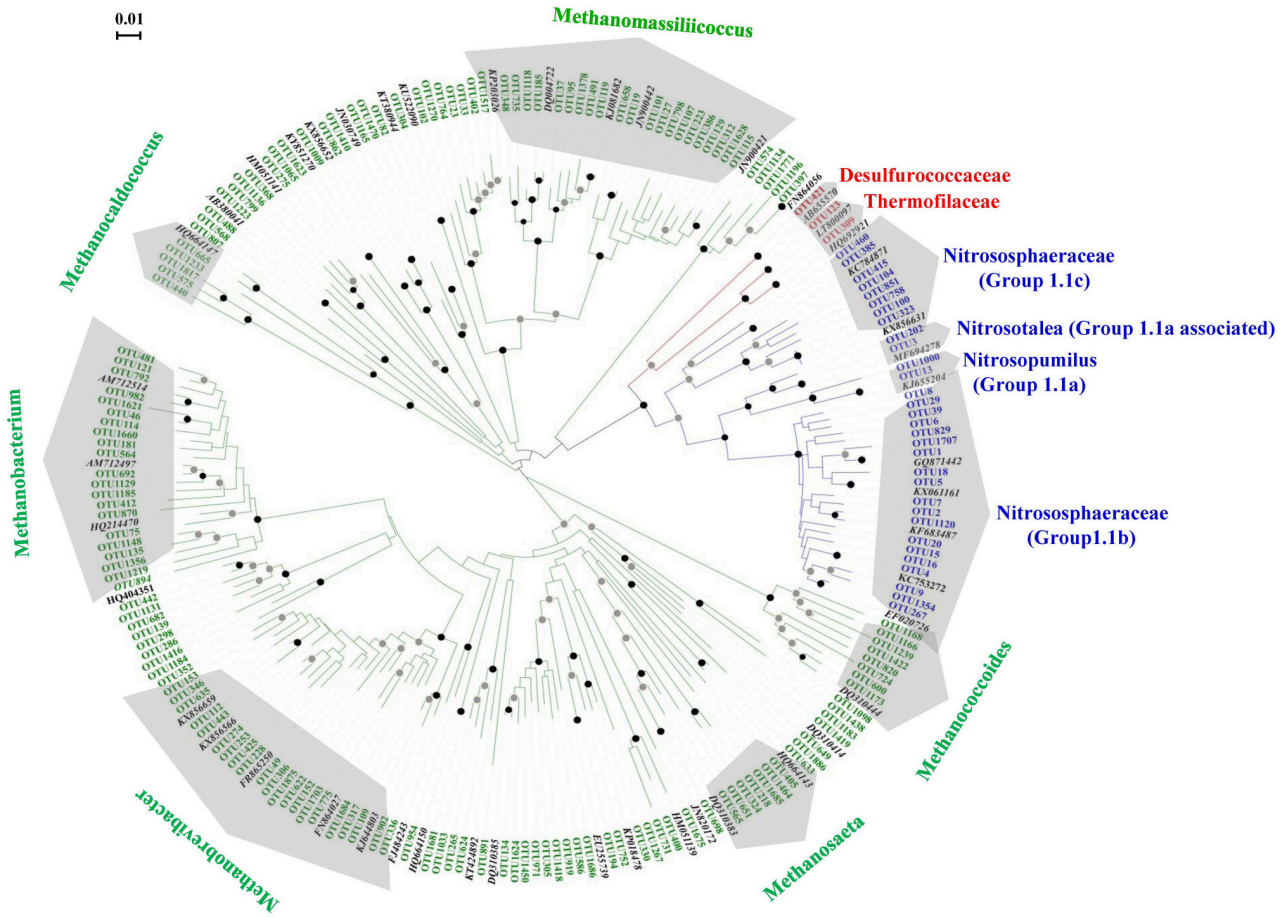
Unrooted phylogenetic tree showing the archaeal OTUs obtained in the black soils with reference clones deposited in the GenBank. The OTU number marked with red, green, and blue letter represented the clones of *Crenarchaeota, Euryarchaeota*, and *Thaumarchaeota*, respectively. The reference archaeal clones marked with black letters. The black and gray circles indicate internal nodes with higher than 90 and 50% bootstrap support, respectively.

In total, 185 archaeal OTUs were detected in this study, and the OTU number ranged from 41 to 130 (Table S1). Among the 185 OTUs, 3, 31, and 151 were found within the phyla *Crenarchaeota, Thaumarchaeota* and *Euryarchaeota*, respectively. Figure 1 shows the phylogenetic tree of the OTUs observed in this study, which indicated that the diversity of *Euryarchaeota* was the highest archaeal phylum in the black soils. The relative abundances, taxonomy information and represented sequences of all the OTUs are shown in Table S3. The closest relative of each OTU identified by BLAST search are shown in Table S4.

The phylogenetic positions of the 31 OTUs with the sequence numbers within *Thaumarchaeota* are shown in Figure S3. Two, 2, 8, and 19 OTUs fell into group 1.1a, group 1.1a-associated, group 1.1c, and group 1.1b, respectively. The sequences belonging to group 1.1b were the most abundant, accounting for 92.35% of the sequences of *Thaumarchaeota*, followed by group 1.1a-associated (6.93%), group 1.1a (0.59%), and group 1.1c (0.13%). Seven OTUs (OTU1~OTU6, OTU1120) were abundant members with total sequences accounting for 80.33% of the *Thaumarchaeota* sequences.

### Linking Environmental Variables and the Distribution Patterns of Archaeal Taxa

**Figure 2:**
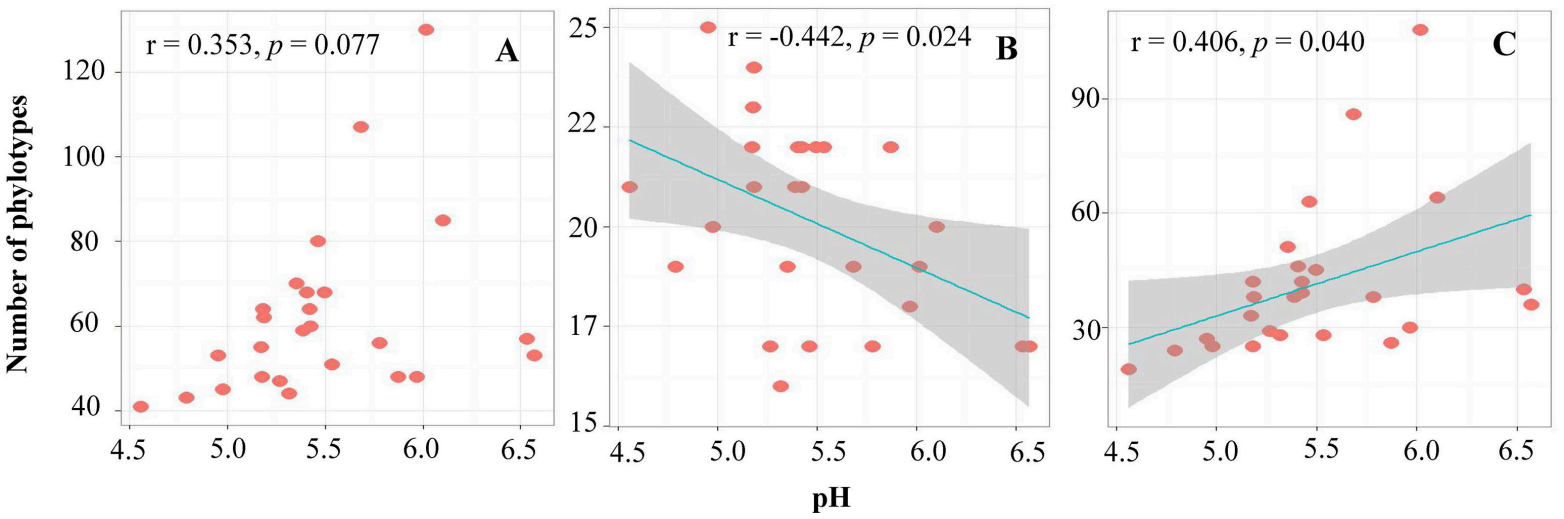
The relationship between soil pH and OTU numbers of total archaea **(A)**, *Thaumarchaeota* **(B)**, and *Euryarchaeota* **(C)**.

Pairwise correlation analysis showed that the alpha-diversity (OTU number) of the archaea had no relationship with any soil parameters. However, the OTU number within the phyla *Thaumarchaeota* and *Euryarchaeota* showed negative (*r* = −0.442, *p* = 0.024) and positive (*r* = 0.406, *p* = 0.040) correlations with the soil pH, respectively (Figure 2).

The relationship between the sampling latitude, soil properties and relative abundance of archaea at the different taxonomy levels was examined using pairwise correlation analysis (Table 1). None of the three phyla had a significant relationship with any environmental variables detected. At the order, genus and OTU levels, only the members belonging to *Thaumarchaeota* had significant correlations with some environmental variables. For example, the relative abundance of *Nitrososphaerales* was positively correlated with the sampling latitude, soil pH, TC, and TN concentrations, while the relative abundance of *Thaumarchaeota*_norank exhibited the reverse tendency. At the genus level, group 1.1a-associated and group 1.1 showed an opposite tendency in their change with the sampling latitude, soil pH, total C and total N concentrations. At the OTU level, 17 OTUs were significantly correlated with the environmental variables. Among these, the abundant members of OTU1 and OTU5 correlated positively, while OTU3 correlated negatively with the sampling latitude; OTU1120, OTU2, and OTU3 were negatively correlated, while OTU6 positively correlated with the soil pH; in addition, OTU1 also showed a significant positive relationship with the total C, total N and soil moisture concentrations, while OTU3 showed the opposite trend (Table 1).

**Table 1:** The relationships between soil physicochemical properties and the relative abundance of archaea at phylum, order, genus, and OTUs level in black soils using Pearson's correlation.

**Taxa**	**Latitude**	**pH**	**TC**	**TN**	**C/N**	**H_**2**_O%**	**TP**	**AK**	**AP**	**$\text{N}{\text{H}}_{4}^{+}$-N**	**$\text{N}{\text{O}}_{3}^{-}$-N**
**PHYLUM**											
*Crenarchaeota*	−0.327	0.227	−0.288	−0.275	−0.130	−0.022	−0.286	−0.216	−0.242	−0.106	−0.049
*Euryarchaeota*	−0.181	−0.129	−0.006	0.021	−0.053	0.249	−0.080	−0.321	−0.298	−0.189	−0.194
*Thaumarchaeota*	0.221	0.079	0.054	0.028	0.071	−0.223	0.121	0.330	0.313	0.191	0.186
**ORDER**											
*Methanobacteriales*	−0.244	−0.234	−0.122	−0.089	−0.126	0.105	−0.146	−0.303	−0.226	−0.172	−0.127
*Methanococcales*	−0.071	0.066	0.036	−0.019	0.252	0.217	−0.248	−0.325	−0.274	−0.072	−0.143
*Methanomassiliicoccales*	−0.062	−0.181	0.082	0.095	0.063	0.213	−0.006	−0.299	−0.285	−0.189	−0.255
*Methanosarcinales*	−0.355	0.324	−0.104	−0.095	−0.082	0.169	−0.113	−0.142	−0.116	−0.112	−0.082
*Nitrososphaerales*	0.632^**^	0.500^**^	0.510^**^	0.482^*^	0.195	0.296	0.248	0.433^*^	0.068	0.181	0.029
*Thaumarchaeota*_norank	−0.566^**^	−0.590^**^	−0.499^**^	−0.478^*^	−0.169	−0.389^*^	−0.193	−0.348	0.027	−0.125	0.036
*Thermococcales*	0.404^*^	−0.131	0.404^*^	0.436^*^	−0.038	0.549^**^	0.286	0.026	−0.234	0.042	0.005
**GENUS**											
*Methanobacterium*	−0.383	−0.258	−0.240	−0.214	−0.118	−0.056	−0.256	−0.331	−0.189	−0.171	−0.123
*Methanobrevibacter*	0.314	−0.027	0.298	0.331	−0.054	0.499^**^	0.263	−0.034	−0.199	−0.073	−0.080
*Methanomassiliicoccus*	−0.062	−0.181	0.082	0.095	0.063	0.213	−0.006	−0.299	−0.285	−0.189	−0.255
*Methanosaeta*	−0.327	0.335	−0.057	−0.047	−0.080	0.216	−0.067	−0.118	−0.100	−0.105	−0.080
*Methanothermococcus*	0.370	−0.135	0.273	0.227	0.228	0.289	0.041	−0.229	−0.234	0.006	−0.055
*Nitrososphaera* (Group1.1c)	0.366	−0.233	0.337	0.343	0.033	0.491^*^	0.306	−0.081	−0.062	0.029	−0.011
*Nitrosotalea* (Group 1.1a associated)	−0.566^**^	−0.590^**^	−0.499^**^	−0.478^*^	−0.169	−0.389^*^	−0.193	−0.348	0.027	−0.125	0.036
*Nitrososphaera* (Group 1.1b)	0.625^**^	0.504^**^	0.503^**^	0.475^*^	0.194	0.286	0.243	0.434^*^	0.069	0.180	0.029
**OTU**											
OTU267 (Group 1.1b)	0.065	−0.347	−0.126	−0.123	−0.025	0.021	−0.136	−0.285	−0.269	0.096	0.129
OTU7 (Group 1.1b)	−0.287	0.723^**^	−0.051	0.030	−0.307	−0.231	0.062	0.228	0.087	−0.162	−0.182
OTU8 (Group 1.1b)	0.640^**^	−0.220	0.660^**^	0.638^**^	0.150	0.719^**^	0.420^*^	−0.036	−0.151	−0.017	−0.087
OTU15 (Group 1.1b)	−0.431^*^	−0.495^*^	−0.380	−0.372	−0.07	−0.357	0.032	−0.251	−0.009	−0.187	−0.099
OTU39 (Group 1.1b)	0.336	−0.060	0.343	0.386	−0.088	0.446^*^	0.356	0.057	−0.087	−0.035	−0.012
**OTU1120** (Group 1.1b)	0.216	−0.610^**^	0.051	0.008	0.171	0.189	0.036	−0.337	−0.096	0.175	0.148
**OTU1** (Group 1.1b)	0.596^**^	0.061	0.598^**^	0.574^**^	0.141	0.436^*^	0.170	0.081	−0.294	−0.024	−0.146
**OTU2** (Group 1.1b)	0.226	−0.620^**^	0.108	0.096	0.018	0.235	0.073	−0.097	0.146	0.288	0.384
**OTU4** (Group 1.1b)	0.121	−0.006	−0.221	−0.271	0.235	−0.232	−0.242	0.154	0.139	0.287	0.220
**OTU5** (Group 1.1b)	0.470^*^	−0.224	0.272	0.202	0.345	0.337	0.005	−0.077	−0.235	0.123	0.001
**OTU6** (Group 1.1b)	−0.375	0.772^**^	−0.211	−0.146	−0.294	−0.236	−0.051	0.149	0.091	−0.166	−0.147
OTU9 (Group 1.1b)	0.280	0.195	−0.049	−0.103	0.218	−0.173	−0.034	0.313	0.300	0.083	0.116
OTU16 (Group 1.1b)	0.072	0.395^*^	0.548^**^	0.589^**^	−0.127	0.433^*^	0.328	0.377	0.058	0.050	0.043
OTU18 (Group 1.1b)	0.518^**^	0.156	0.862^**^	0.855^**^	0.092	0.817^**^	0.586^**^	0.177	−0.061	0.021	−0.049
OTU1354 (Group 1.1b)	−0.313	0.661^**^	−0.169	−0.227	0.263	−0.384	0.104	0.452^*^	0.424^*^	−0.145	−0.196
OTU1707 (Group 1.1b)	−0.086	−0.256	−0.005	−0.061	0.246	−0.040	−0.304	−0.132	−0.316	−0.095	−0.187
**OTU3** (Group 1.1a associated)	−0.566^**^	−0.590^**^	−0.499^**^	−0.479^*^	−0.168	−0.390^*^	−0.193	−0.348	0.027	−0.125	0.035

TC, TN, and TP represent soil total carbon, nitrogen and phosphorus, respectively. AK and AP represent soil available potassium and phosphorus, respectively. Correlations with significant values (

* p < 0.05;

** *p < 0.01) are shown in bold. The bold OTUs are the most abundant OTUs in the black soils*.

### Archaeal Community Structure

**Figure 3:**
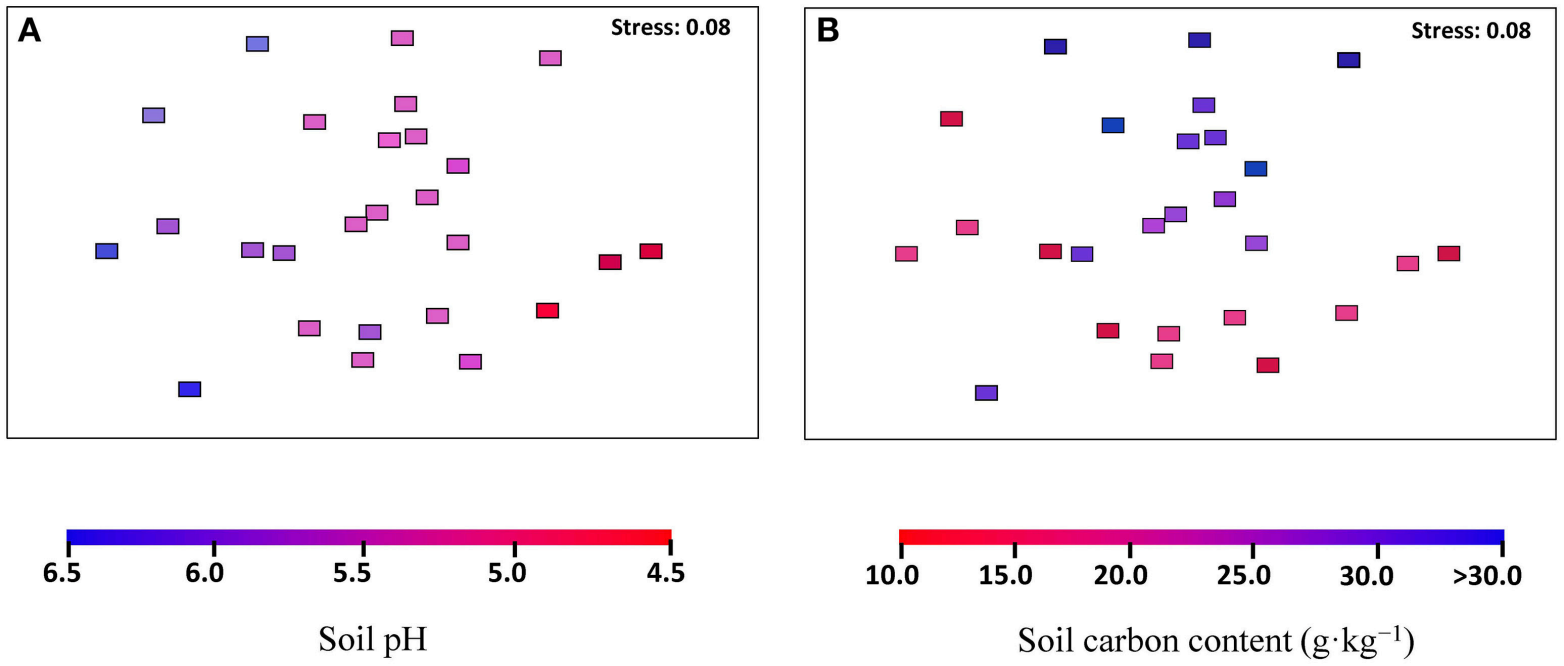
Archaeal community compositions in the black soils, as indicated by non-metric multi-dimensional scaling plots of weighted pairwise UniFrac community distances between sites. Sites are color-coded to soil pH gradient **(A)** and soil total carbon gradient **(B)**.

**Figure 4:**
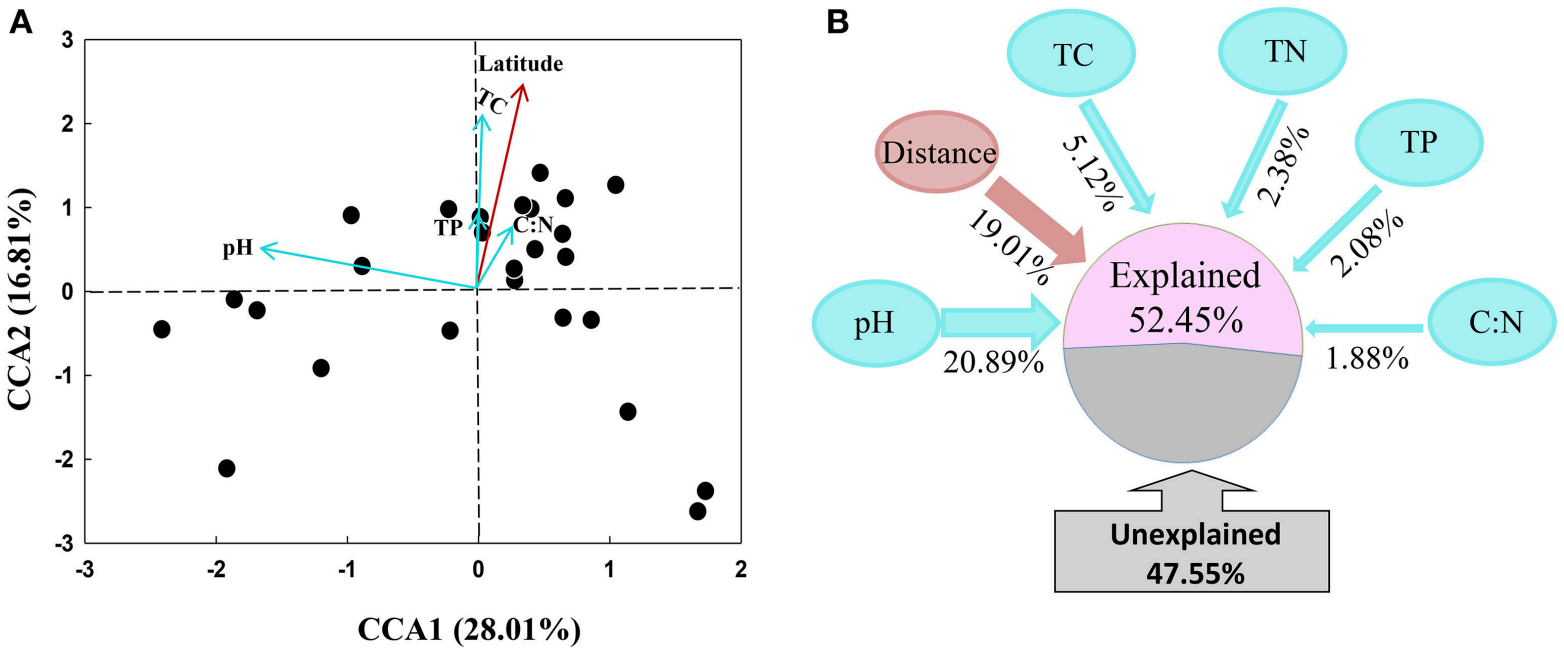
Canonical correspondence analysis (CCA) of archaeal communities and environmental factors **(A)**, and variation partition analysis of spatial distance and soil variables on the explanation of variations of archaeal community structures **(B)**.

The archaeal community structures were illustrated using weighed NMDS plots based on the gradient of the soil pH and the soil total C content (Figure 3). The NMDS1 and NMDS2 axis score was negatively correlated with the soil pH (r = −0.836, *p* < 0.001) and positively correlated with the soil total C (r = 0.649, *p* < 0.001), respectively (Table S5). The CCA analysis indicated that the archaeal community structures differed among the sampling locations (Figure 4). CCA1 and CCA2 explained 28.01 and 16.81% of the community structures, respectively. Among the environmental factors examined, the soil pH was the dominant factor in shifting community structures along the CCA1 axis, while the latitude of the sampling locations was the dominant factor driving the changes of communities along the CCA2 axis. In addition, a VPA analysis showed that the geographic distances explained 19.01% of the community variation, and the selected soil parameters explained 32.35% with soil pH being the master variable explaining 20.89% of the community variation, leaving 47.55% of the variation unexplained.

## Discussion

### The Abundance of Archaea Across Black Soils

In this study, compared with the data of bacterial abundance reported previously (Liu et al., 2016a), the ratio of archaeal to bacterial 16S rRNA gene copies ranged from 0.032 to 0.085, indicating that the archaeal abundance accounted for 3.11–7.80% of the total prokaryotes in the black soils. The abundance of the archaea in the agricultural soils varied between studies. Bates et al. (2011) used universal primers 515f/806r for HTS sequencing and found that the archaea comprised 2% of all the sequences with a range from 0 to >10% in individual soils. Bengtson et al. (2012) reported that the ratio of archaeal to bacterial 16S rRNA gene copy numbers ranged from 0.02% at low pH values to more than 7% at pH 8 in the Hoosfield acid strip at Rothamsted Research, UK. Cao et al. (2012) also reported that archaea accounted for 0.20–9.26% of the total prokaryotes in the selected Chinese soils. Therefore, the archaeal abundance across black soil zoon of northeast China is consistent with above-mentioned studies.

The influence of soil pH on the abundance of the archaea varied with the studies. Tripathi et al. (2013) detected the archaeal abundance to be negatively correlated with the soil pH across 27 soil samples collected from tropical forest and non-forest sites. However, Bengtson et al. (2012) found a strong positive relationship between the archaeal abundance and the soil pH in the temperate region. The soil samples used for this study were collected from the temperate region, and our finding is consistent with that of Bengtson et al. (2012). In addition to the soil pH, the soil organic C and total N were positively correlated with the archaeal abundance in several selected Chinese soils (Cao et al., 2012), which partially agreed with the findings of this study, showing that the soil C, N, and P concentrations, available K concentration and soil moisture content were highly positively correlated with the archaeal abundance (Figure S2). This finding suggests that there are multiple soil factors controlling the population of archaea in the black soils.

### The Composition of Archaea in the Black Soils

*Thaumarchaeota* was the dominating archaeal phylum in the black soils, which is consistent with the findings of Tripathi et al. (2015) who investigated 24 samples of topsoil collected from a tropical biome and 25 soils from a temperate biome. Our results are also consistent with the findings of Shi et al. (2016) who found that *Thaumarchaeota* was the most abundant phylum in the soils of eastern Tibetan Plateau. However, at the lower taxonomic level, *Thaumarchaeota* group 1.1b was the most abundant in the black soils (average of 87.23% of the total archaeal sequences), while in the results of Tripathi et al. (2015), *Thaumarchaeota* group 1.1b accounted for 48.7% of all the archaeal sequences, but the proportion of group 1.1b in Tibetan Plateau was not reported (Shi et al., 2016). This finding indicated that the members of *Thaumarchaeota* group 1.1b were the overwhelming archaea in the arable black soils. Recently, Lu et al. (2017) showed that the relative abundance of *Thaumarchaeota* group 1.1b was higher in the agricultural soils than in the forest soils.

Considering group 1.1b of *Thaumarchaeota* was taxonomically classified as *Nitrososphaera* (Bomberg, 2016), the relative abundance of *Nitrososphaera* (group 1.1b) in this study was comparable to the data observed in our former study of the AOA, in which the *Nitrososphaera* cluster and the *Nitrososphaera* sister cluster together comprised 89.34% of the AOA sequences across the same soil samples (Liu et al., 2018). Thus, we hypothesized that the majority of archaea in black soils contribute to nitrification.

*Thaumarchaeota* group 1.1a and group 1.1a-associated were phylogenetically classified into *Nitrosopumilus* and *Nitrosotalea*, respectively (Table S2; Bomberg, 2016). The findings of this study and our previous study of the AOA (Liu et al., 2018) indicated that those groups were minor, and the relative abundances of those groups are comparable between the two studies. For example, the relative abundance of the *Nitrosospumilus* cluster and *Nitrosotalea* comprised averages of 1.98 and 8.68% of the AOA sequences, respectively (Liu et al., 2018). Similarly, *Thaumarchaeota* groups 1.1a and 1.1a-associated accounted for averages of 0.54 and 6.23% of the archaeal sequences in this study (Table S2). The consistent results between the two studies suggest that the results observed in this study were correct and that the primer set used was suitable for analysis of the archaeal communities at least in the black soils.

*Thaumarchaeota* group 1.1c was frequently detected in acidic agricultural soils (Lehtovirta et al., 2009) and acidic forest soils (Jurgens et al., 1997; Bomberg and Timonen, 2009; Weber et al., 2015). However, in this study, this group only accounted for an average of 0.12% of the total archaeal sequences (Table S2), suggesting that group 1.1c is a rare member of the archaea in the arable black soils, even though the pH of most of the soils used for this study was below 6.0 (Table S1). Unlike *Thaumarchaeota* groups 1.1a and 1.1b, the phylogenetic position of group 1.1c was unclear, since no representative of this group has yet been obtained in pure culture (Weber et al., 2015; Bomberg, 2016). A recent study showed that group 1.1c are heterotrophic archaea, and its growth was not related to ammonia oxidation (Weber et al., 2015).

Although the relative abundance of *Euryarchaeota* only comprised 5.75% of the total sequences (Table S2), the OTU number within this phylum was greater than that in the *Thaumarchaeota* (Figure 1), suggesting that the diversity of *Euryarchaeota* is at the highest level among the archaeal phylum in the black soils. An interesting finding within the *Euryarchaeota* is that the majority of sequences are related to the methanogenic archaea (Table S2). Methanogenic archaea are usually considered to exist in highly reduced and anoxic conditions, such as wetlands, paddy fields, marine, or lake sediments, and the intestinal tracts of human and animals (Garcia et al., 2000; Liu and Whitman, 2008). However, the samples used in this study were collected from upland arable soils, where methane emission is not thought to be an important ecological process. We hypothesize that the diversity of the methanogenic archaea is related to the anaerobic conditions in localized micro sites, anaerobic seasonal conditions, or due to land conversion. A large number of OTUs grouped into the methanogenic *Euryarchaeota* were also found in upland soils across from South and North China (Hu et al., 2013).

In contrast to the *Euryarchaeota*, only 31 OTUs of *Thaumarchaeota* were observed across the black soils. Among these, seven OTUs were overwhelmingly dominant (Table S3), which suggested that the dominating members of archaea in the black soils are simplified. This finding is consistent with the observations of Bates et al. (2011) that two OTUs (DSC1 and DSC2) were highly abundant across 146 soils obtained globally and also agrees with the findings of Tripathi et al. (2013), who detected 6 OTUs as the most abundant archaea in 27 tropical soils.

### Effects of Environmental Factors on Alpha Diversity of Archaeal Communities in the Black Soils

Archaeal alpha diversity was very low in comparison with the bacterial diversity in the same black soil samples (Liu et al., 2014). Lower diversities of soil archaeal communities were also reported in other studies (Auguet et al., 2010; Singh et al., 2012; Tripathi et al., 2013). Soil pH was commonly regarded to be the major factor in the determination of the soil bacterial diversity (Fierer and Jackson, 2006; Chu et al., 2010). Similarly, several studies also indicated that the alpha diversity of the archaea in tropical and temperate soils was negatively correlated with the soil pH (Tripathi et al., 2013, 2015). In contrast, the archaeal alpha diversity in the soils from the Tibetan Plateau was significantly negatively correlated with the soil moisture, total N, total organic C (*p* < 0.01), total dissolved N, NH${}_{4}^{+}$-N and NO${}_{3}^{-}$-N but not with the soil pH (Shi et al., 2016). Furthermore, Singh et al. (2012) showed that the archaeal diversity in Mt. Fuji soils had a strong positive correlation with the soil NH${}_{4}^{+}$-N, K and NO${}_{3}^{-}$-N. In this study, we found that the OTU richness of the archaea had no relationship with any of the soil properties measured, but the OTU richness within *Thaumarchaeota* and *Euryarchaeota* was slightly correlated with soil pH (Figure 2). This change in the tendency of the OTU richness of *Thaumarchaeota* is not consistent with the finding of Lu et al. (2017), who detected that the OTU richness of *Thaumarchaeota* in forest and cropping soils had no relationship with soil pH. However, the finding of the OTU richness of *Euryarchaeota* in this study was supported by the results of Hu et al. (2013) who found that the OTU richness of *Euryarchaeota* was significantly positively correlated with the soil pH in both the uplands and paddy soils (*P* < 0.001). Thus, the effects of environmental factors on the alpha diversity of archaea are very complex and require further investigation.

### Effects of Environmental Factors on the Distribution of Archaeal Taxonomy

Within the most abundant phylum *Thaumarchaeota*, the responses of *Nitososphaerales* and *Thaumarchaeota*_norank at the order level, and group 1.1a-associated and group 1.1b at the genus level to sampling latitude, soil pH, total C, total N were the opposite (Table 1). This finding suggests the physiological and ecological characteristics of the different taxa of *Thaumarchaeota* varied greatly. Recently, Lu et al. (2017) observed that the relative abundance of *Thaumarchaeota* SCG (group 1.1b) and TG (group 1.1c) was positively and negatively correlated with soil pH, respectively. The tendency of the change of group 1.1b is similar to that in this study, while the relative abundance of group 1.1c in the black soil showed no significant relationship with soil pH (Table 1). The lack of a significant change of group 1.1c with the soil pH in black soils could be related to its very low abundance (average 0.12%), but it accounted for ~25% of the *Thaumarchaeota* sequences in the study of Lu et al. (2017).

The relative abundances of the seven most abundant OTUs of *Thaumarchaeota* responded strongly to a number of environmental variables (Table 1). Three OTUs (OTU1, 3 and 5) were significantly correlated to the sampling latitudes, suggesting that the biogeographic distribution of archaea along the latitude exist in the black soil region. Additionally, three OTUs (OTU2, 3 and 1120) are negatively and OTU6 positively correlated with the soil pH, suggesting that the different OTUs are niche-specialized for growth at various pH levels (Tripathi et al., 2013). This finding supports the result that the soil pH is an important ecological niche to determine not only the AOA distribution (Nicol et al., 2008; Gubry-Rangin et al., 2011) but also the archaeal distribution (Tripathi et al., 2013, 2015).

### Biogeographic Distribution of Archaeal Communities in the Black Soils

The biogeographic distribution of the archaeal communities at a large scale is controversial. Ma et al. (2017) investigated the biogeographic patterns of soil bacterial, fungal, and archaeal communities across four vegetation forest zones in Eastern China, and they found that geographic distance had no influence on the archaeal communities, while the bacterial and fungal communities showing a distance-decay pattern. Similarly, (Liu et al., 2016b) also indicated that the archaeal community compositions in the lake sediments of Tibetan Plateau were not correlated with the geographic distance or altitude. Zhang et al. (2018) recently found that the bacterial, archaeal and methanogenic communities in the Chinese wetlands from all different regions did not show a significant distance-decay pattern. In contrast, Shi et al. (2016) revealed that the soil archaeal communities in the Tibetan Plateau displayed a strong distance-decay. In this study, similar to the findings of bacterial communities in the same soil samples (Liu et al., 2014), we also observed that the archaeal communities were geographically distributed across the black soil zone. However, the geographic distance on the explanation of the archaeal and bacterial community distribution was difference between two studies. The geographical distance explained 19% of the variation of the archaeal communities, which was larger than that of the bacterial communities (14.75%) (Liu et al., 2014) but was comparable to the AOA communities (17.01%) (Liu et al., 2018). Since the majority of archaea (*Thaumarchaeota* group 1.1b and group 1.1a) obtained in this study were phylogenetically grouped to be AOA (Nicol and Schleper, 2006), the biogeographic distribution of the archaeal communities in the black soils, to some extent, was somewhat consistent with the AOA communities as we reported previously (Liu et al., 2018).

Similar to the findings of the biogeographical distribution of the soil bacterial communities (Lauber et al., 2009; Rousk et al., 2010; Griffiths et al., 2011), pH has been shown to be the dominating soil factor in driving archaeal communities in various ecosystems (Cao et al., 2012; Hu et al., 2013; Tripathi et al., 2013, 2015). In contrast, (Liu et al., 2016b) found that the sediment salinity was the sole factor in determining archaeal communities across lake sediments in the Tibetan Plateau. In comparison, Zheng et al. (2013) reported that the soil pH, sample depth and longitude played a key role in shaping archaeal distribution in the non-flooded soil habitat, while sampling depth, longitude and NH${}_{4}^{+}$-N were the most important factors in the flooded soil habitat. Recently, Zhang et al. (2018) found that the biogeographic patterns of the archaeal communities along the latitudinal gradient in the Chinese wetland soils were driven by multiple factors, including the soil pH, total organic carbon, total phosphorus, mean annual temperature, annual frost days, mean annual precipitation, and direct solar radiation. Our study showed that the distribution patterns of some archaeal taxa were significantly correlated with some soil factors (Table 1), and the soil pH was the dominant factor (contributed 20.89% variation) in the determination of the archaeal community distribution across the black soils. This finding is consistent with our previous studies showing that the soil pH was the key edaphic factor in the determination of the bacterial and AOA communities in the same soil samples (Liu et al., 2014, 2018).

In conclusion, this study revealed that the abundance of archaea in the black soils occupied 3.11~7.80% of the total prokaryotes. Multiple soil factors were found to regulate the archaeal abundance in the black soils that differed from bacteria and fungi (Liu et al., 2015; Liu et al., 2016b). Among the three observed phyla, *Thaumarchaeota* was the dominant archaea, followed by *Euryarchaeota*, and *Crenarchaeota* was occasionally observed at a low abundance. This study also suggests that the composition of the archaeal communities (with only 185 OTUs observed) is simplified compared to the bacterial or fungal communities in the same soils. The opposite effects of the environmental factors on the dominant OTUs suggest that the physiological characteristics of the different archaeal members are diversified in the black soils. Similar to the bacterial communities, the archaeal communities across the black soil zone are also geographically distributed with the soil pH being the major determining soil factor. In addition, *Thaumarchaeota* group 1.1b (*Nitrososphaera*) was the dominant genus, suggesting that the majority of archaea play important roles in nitrogen cycling in the upland black soils.

## Author Contributions

GW and JL conception of the study. JL, GW, QY, and YYS designed the experiments. ZY, QY, and YS performed the experiments. QY and ZY interpretation of the results. GW, and JL wrote the manuscript. HC, JJ, XL, CT, and AF revised the manuscript.

### Conflict of Interest Statement

The authors declare that the research was conducted in the absence of any commercial or financial relationships that could be construed as a potential conflict of interest.

## Supplementary Material

Figure S1 A map of sampling locations across black soil zone of northeast China.

Figure S2 The linear relationships between archaeal abundance and soil pH **(A)**, soil moisture **(B)**, soil available potassium content **(C)**, soil total carbon content **(D)**, soil total nitrogen content **(E)**, and soil phosphorus content **(F)**.

Figure S3 Neighbor-joining tree showing the phylogenetic positions of *Thaumarchaeota* OTUs observed in the black soils. The numbers in the parentheses after individual OTUs indicate the observed number of reads, the numbers in the parentheses of reference clones, or isolates indicate the accession numbers in the NCBI website. Bootstrap values < 50 are not shown. The scale bar represents 0.05 substitutions per nucleotide.

Table S1 Location, physicochemical properties, abundance, OTU richness and phylogenetic diversity of archaeal community in 26 black soils for this study.

Table S2 Relative abundance (%) of archaeal community at phylum, order and genus levels across all soil samples.

Table S3 Relative abundance, taxonomy information and represented sequence of OTUs observed in the black soils. The bold OTUs are the most abundant OTU in the black soils.

Table S4 The closest relative of each OTU observed in the black soils.

Table S5 The correlation coefficients (r) and significance levels (P) of pairwise 581 correlation between NMDS scores and soil total C content, and soil pH value. Values 582 in bold indicate significant correlations (P < 0.01).
