# Supplementary figures and images for: Biogeographic Distribution Patterns of the Archaeal Communities Across the Black Soil Zone of Northeast China
Source: Front Microbiol. 2019 Jan 25;10:23. doi: 10.3389/fmicb.2019.00023 (PMC6355713; doi:10.3389/fmicb.2019.00023)

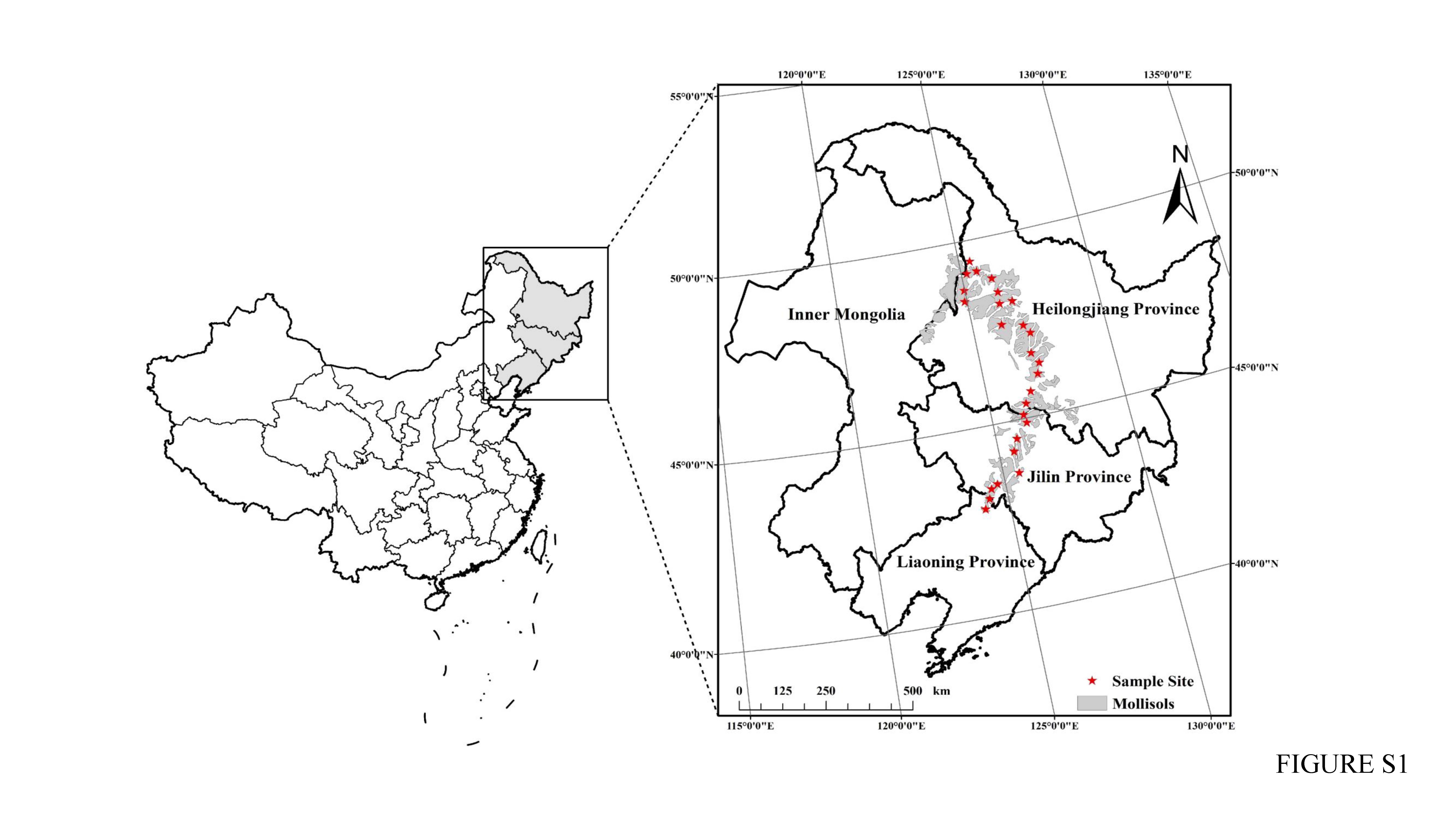

Supplement: Figure S1 — A map of sampling locations across black soil zone of northeast China. [file Image_1.JPEG]

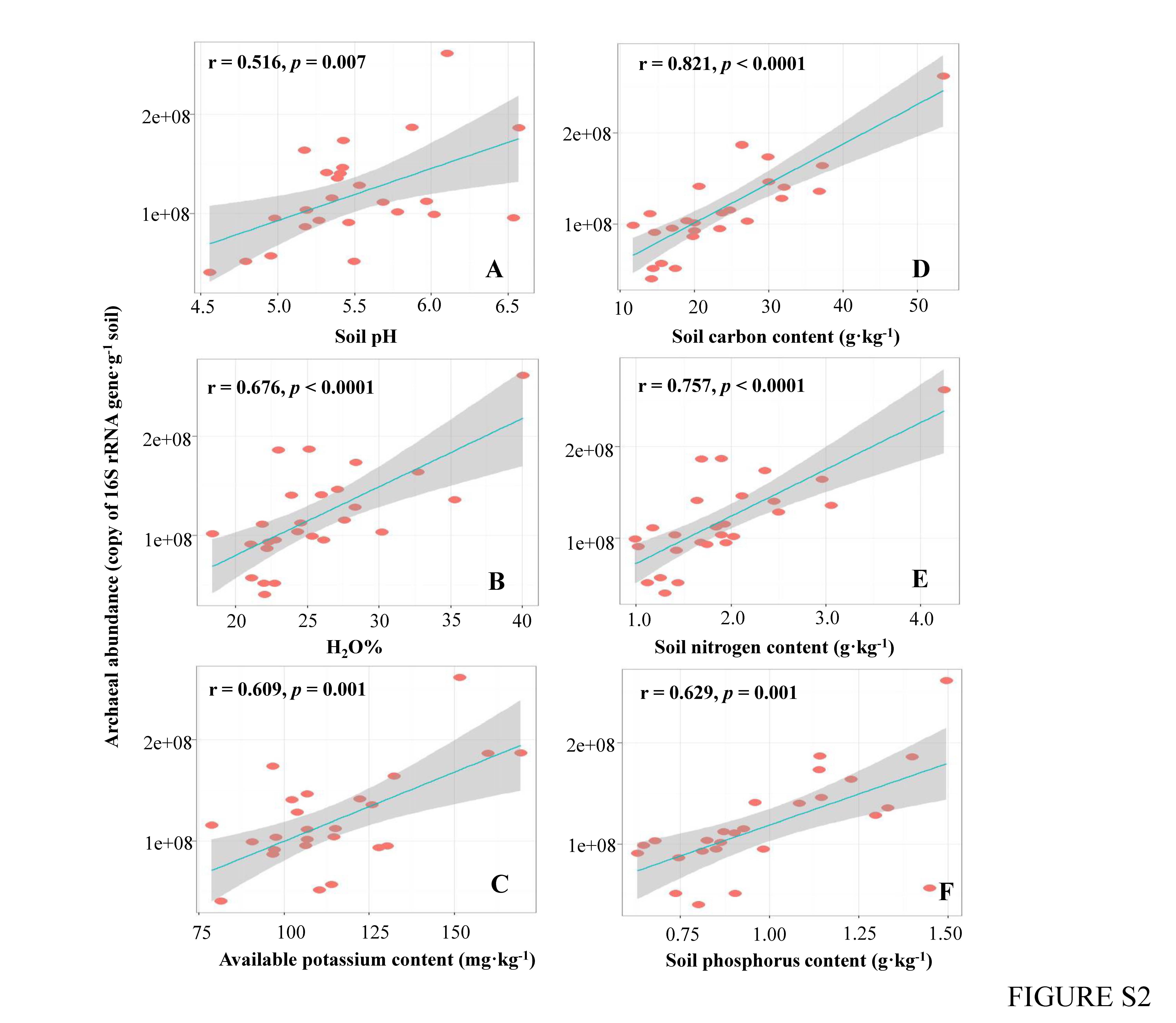

Supplement: Figure S2 — The linear relationships between archaeal abundance and soil pH (A), soil moisture (B), soil available potassium content (C), soil total carbon content (D), soil total nitrogen content (E), and soil phosphorus content (F). [file Image_2.JPEG]

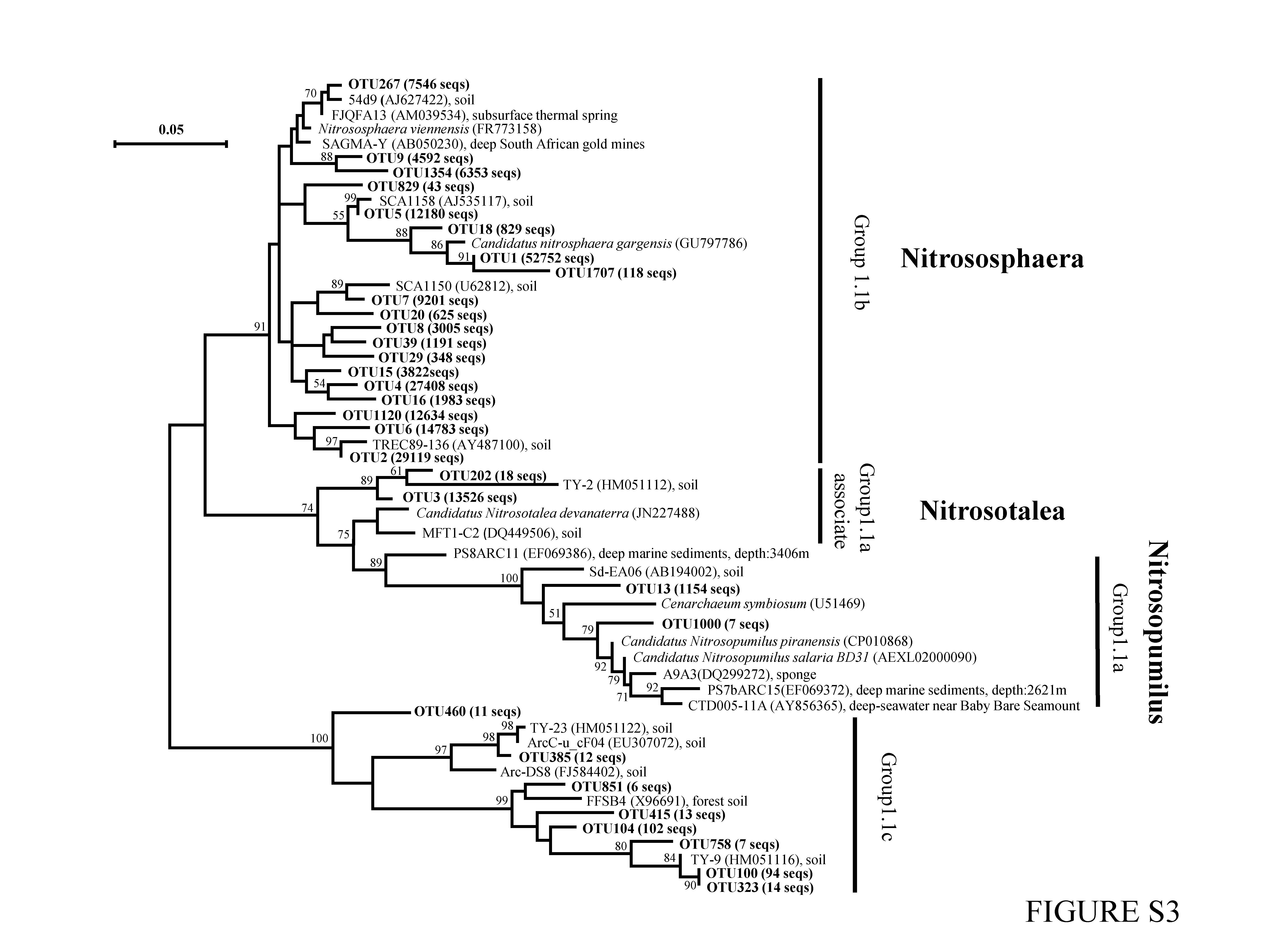

Supplement: Figure S3 — Neighbor-joining tree showing the phylogenetic positions of Thaumarchaeota OTUs observed in the black soils. The numbers in the parentheses after individual OTUs indicate the observed number of reads, the numbers in the parentheses of reference clones, or isolates indicate the accession numbers in the NCBI website. Bootstrap values < 50 are not shown. The scale bar represents 0.05 substitutions per nucleotide. [file Image_3.JPEG]
