# Supplementary material for: Biogeographic Distribution Patterns of the Archaeal Communities Across the Black Soil Zone of Northeast China
Source: Front Microbiol. 2019 Jan 25;10:23. doi: 10.3389/fmicb.2019.00023 (PMC6355713; doi:10.3389/fmicb.2019.00023)
Supplement: Table S4 — The closest relative of each OTU observed in the black soils. [file Table_4.DOC]

**Table S4** The closest relative of each OTU observed in the black soils.

| OTU ID | Closest relatives | | | Identity (%) | Alignment |
| --- | --- | --- | --- | --- | --- |
|  | Clone | Source | Accession number |  |  |
| *Crenarchaeota* | | | | | |
| 421 | A0610D002_M02 | Rice paddy soil | AB655570 | 100 | 385/385 |
| 123 | Arc16S_1 | Rice paddy soil | KY851203 | 100 | 385/385 |
| 309 | CS-192-16 | Paddy soil | HQ692921 | 100 | 385/385 |
| *Euryarchaeota* | | | | | |
| 19 | LS3-73-162 | Cinnamon soil | JN900442 | 99 | 385/386 |
| 23 | UT11 | Field soil | KC784845 | 100 | 385/385 |
| 27 | CONTROL7_ARCH2E12 | Soil | HF952227 | 100 | 385/385 |
| 33 | CS45-53 | Forest soil | KJ632787 | 100 | 385/385 |
| 37 | MTG-45-162 | Cinnamon soil | JN900420 | 99 | 383/385 |
| 46 | Sv-30 | High arctic permafrost peat | AM712514 | 96 | 369/385 |
| 49 | IU-FSC-2c_Ar-A047 | Soil | AB380038 | 99 | 385/386 |
| 75 | LW-26 | Subglacial sediment | HQ214470 | 95 | 366/385 |
| 82 | SH-LY0 | Coalbed water | KT380944 | 100 | 386/386 |
| 95 | Elev_16S_arch_1578 | Rhizosphere soil | EF022241 | 99 | 383/385 |
| 101 | DZ3-10B-A-7 | Qilian mountain | KJ834181 | 99 | 382/385 |
| 102 | Elev_16S_arch_1848 | Rhizosphere soil | EF022496 | 99 | 382/385 |
| 107 | GYnA-101 | Red soil | KP018433 | 99 | 384/385 |
| 109 | Sto-0_6-rtC02 | Storegga Slide sediment, 0-6 cm depth | GU055850 | 97 | 374/386 |
| 112 | Wet83 | Wetland soil | KX856659 | 96 | 378/396 |
| 114 | JQ762566 | Wetland soil in Lake BangongCo | JQ762566 | 96 | 363/386 |
| 115 | MTG-47-162 | Cinnamon soil | JN900421 | 99 | 381/385 |
| 119 | lj-arc11 | Copper contaminated soil | KJ081682 | 99 | 384/385 |
| 121 | Sv-30 | High arctic permafrost peat | AM712514 | 95 | 369/387 |
| 129 | 34 | Soil sample from Puguang gas field | JX082285 | 100 | 385/385 |
| 134 | mrR1.35 | Mackenzie River | DQ310385 | 100 | 385/385 |
| 135 | Wet11 | Wetland soil | KX856592 | 97 | 374/385 |
| 139 | ZSA12 | Microbial mats from gold mine | GU205487 | 99 | 381/386 |
| 152 | A0423R002_B20 | Rice paddy soil | AB651067 | 100 | 385/385 |
| 153 | mrR1.57 | Mackenzie River | DQ310389 | 100 | 385/385 |
| 181 | Sv-30 | High arctic permafrost peat | AM712514 | 100 | 385/385 |
| 185 | SSCP band Bo69-06-02 | Arable Europe soil | DQ004722 | 99 | 381/385 |
| 194 | WC-ARC-21 | Freshwater | EU255739 | 100 | 386/386 |
| 218 | 6FA-C4 | Moonmilk deposit on granite wall | HG008888 | 97 | 376/386 |
| 223 | IU-FSC-8_Ar-A070 | Soil | AB380059 | 100 | 385/385 |
| 228 | Upl58 | Upland soil | KX856566 | 96 | 373/387 |
| 253 | MAIZE_39 | Rice field soil | KM463035 | 99 | 384/386 |
| 265 | AR44-1_H11_F | Marine sediment | KT424892 | 92 | 355/387 |
| 274 | Upl52 | Upland soil | KX856560 | 99 | 380/385 |
| 275 | T40UCB9 | Hot spring sediments | MG008504 | 100 | 386/386 |
| 286 | LS24-25-86 | Cinnamon soil | JN900450 | 100 | 385/385 |
| 298 | ZYsA-8 | Yellow-cinnamon soil | KP018426 | 98 | 379/386 |
| 304 | GQ365371 | Groundwater | LN796263 | 100 | 385/385 |
| 305 | IG2G02 | Water | JF835248 | 93 | 365/383 |
| 306 | Upl65 | Upland soil | KX856573 | 99 | 385/386 |
| 312 | ARC2_5_F02 | Groundwater | KC604460 | 99 | 381/385 |
| 317 | MY-24-86 | Cinnamon soil | JN900404 | 96 | 371/386 |
| 324 | RTA-C17 | Biofilm | LN864967 | 98 | 380/386 |
| 330 | QYnA-46 | Nodules separated from brown soil | KP018478 | 97 | 375/386 |
| 336 | REG1522 | Zoige wetland | KJ644803 | 97 | 376/386 |
| 346 | J1105228_C-27_M13F-47 | Paddy soil | KJ604045 | 96 | 370/386 |
| 348 | MTG-69-162 | Cinnamon soil | JN900424 | 99 | 381/385 |
| 352 | mrR1.57 | Mackenzie River | DQ310389 | 97 | 374/386 |
| 368 | ZZ-67 | Fluvo-aquic soil | HM051141 | 97 | 374/386 |
| 386 | W50mCGB752 | Serpentinized dunite | JN002507 | 100 | 385/385 |
| 397 | B235106A02 | River water | FN864056 | 100 | 387/387 |
| 400 | ZZ-64 | Fluvo-aquic soil | HM051139 | 93 | 357/385 |
| 402 | A59P0 | Paddy soil | KP203026 | 99 | 380/385 |
| 405 | OTU_340_915_161 | Microbial mat of cyanobacterial patch | X021808 | 90 | 332/370 |
| 412 | Sv-30 | High arctic permafrost peat | AM712514 | 94 | 364/387 |
| 425 | A0610D002_B07 | Rice paddy soil | AB655662 | 97 | 376/386 |
| 440 | N8-A11 | Neuston of Lake Llebreta | FN691485 | 99 | 385/387 |
| 442 | LJ141_1 | Roots | HQ404351 | 98 | 380/386 |
| 443 | t15d69Ha52 | Rice Field Soil | FR865250 | 97 | 373/384 |
| 481 | Sv-30 | High arctic permafrost peat | AM712514 | 95 | 368/387 |
| 488 | IU-FSC-4_Ar-A011 | Soil | AB380041 | 100 | 385/385 |
| 491 | lj-arc11 | Copper contaminated soil | KJ081682 | 96 | 371/385 |
| 564 | Sv-30 | High arctic permafrost peat | AM712514 | 95 | 365/385 |
| 565 | mrR1.51 | Mackenzie River | DQ310383 | 95 | 365/386 |
| 568 | B112P50 | Paddy soil | KP327900 | 95 | 366/385 |
| 574 | B235106A02 | River water | FN864056 | 93 | 361/388 |
| 575 | Arch37-AMD2012 | Acid mine drainage | KP996646 | 94 | 362/387 |
| 586 | A0610R001_K14 | Rice paddy soil | AB652175 | 96 | 369/386 |
| 600 | mrR2.33 | Mackenzie River | DQ310444 | 97 | 372/385 |
| 622 | A0423R002_B20 | Rice paddy soil | AB651067 | 99 | 382/386 |
| 624 | AR44-1_H11_F | Marine sediment | KT424892 | 93 | 362/389 |
| 633 | AC16Sarch38 | Arsenic contaminated sediment | HQ664143 | 99 | 381/384 |
| 635 | YLA097 | Lake | JF262377 | 96 | 371/386 |
| 649 | AE_21 | Cold freshwater spring | KJ566433 | 95 | 368/386 |
| 651 | C125P100 | Paddy soil | KP327963 | 94 | 361/384 |
| 658 | 138 | Ancient dental calculus of individual in Les Fedons | LN827541 | 99 | 356/359 |
| 665 | AC16Sarch43 | Arsenic contaminated sediment | HQ664147 | 98 | 377/386 |
| 682 | mrR1.32 | Mackenzie River | DQ310381 | 99 | 385/386 |
| 692 | Sv-08 | High arctic permafrost peat | AM712497 | 99 | 381/386 |
| 698 | E03 | Ferromanganese deposit | JN820172 | 99 | 385/386 |
| 724 | TF03 | Ferromanganese deposit | JN820183 | 96 | 370/385 |
| 731 | C1FA2CD04 | Water | GU127390 | 95 | 367/385 |
| 735 | SSCP band Bo64-11-05 | Arable Europe soil | DQ004713 | 99 | 380/385 |
| 752 | ZSA4 | Microbial mats from gold mine | GU205479 | 95 | 366/385 |
| 764 | lj-arc10 | Copper contaminated soil | KJ081681 | 98 | 380/387 |
| 775 | A68 | Moderate saline soil | EU328119 | 97 | 375/386 |
| 792 | Sai_E1_40 | Freshwater sediment | GU257010 | 97 | 358/369 |
| 798 | W50mCGB748 | Serpentinized dunite | JN002503 | 99 | 380/385 |
| 799 | A0610D003_E05 | Rice paddy soil | AB655774 | 97 | 374/385 |
| 807 | AK17 | Bor Khlueng hot spring | AY555816 | 84 | 325/388 |
| 820 | WC-ARC-7 | Water | EU255729 | 98 | 378/385 |
| 862 | Im2 | Termite Incisitermes marginipennis | MF407518 | 100 | 386/386 |
| 870 | ZW-M-3 | Zoige wetland, Tibetan Plateau | KF360009 | 94 | 362/387 |
| 891 | ZSA14 | Microbial mats from gold mine | GU205489 | 97 | 366/378 |
| 894 | LAa02.20 | Water at 2 m | EU782011 | 92 | 354/385 |
| 902 | WC-ARC-5 | Water | EU255727 | 99 | 382/386 |
| 919 | mrR1.04 | Mackenzie River | DQ310410 | 96 | 369/386 |
| 954 | Z17MFA29 | Water | FJ484243 | 94 | 363/388 |
| 971 | QYsA-95 | Brown soil | KP018497 | 100 | 386/386 |
| 982 | Sv-30 | High arctic permafrost peat | AM712514 | 98 | 377/385 |
| 1009 | Wet75 | Wetland soil | KX856652 | 100 | 386/386 |
| 1033 | AR44-1_H11_F | Marine sediment | KT424892 | 93 | 360/387 |
| 1065 | MFC-A56 | Rice MFC anode | JF325932 | 100 | 386/386 |
| 1098 | mrR1.15 | Mackenzie River | DQ310414 | 84 | 317/377 |
| 1118 | HDBA_SITU757 | Hanford Site 300 Area subsurface | HM187574 | 97 | 375/385 |
| 1129 | Sv-08 | High arctic permafrost peat | AM712497 | 97 | 374/384 |
| 1131 | Boil12 | Wetland soil in Lake BangongCo | JQ762561 | 98 | 379/386 |
| 1134 | B235106A02 | River water | FN864056 | 95 | 366/387 |
| 1136 | IMCUGW21A65 | Groundwater samples from Inner Mongolia | JX196240 | 99 | 384/385 |
| 1148 | Wet11 | Wetland soil | KX856592 | 98 | 378/385 |
| 1165 | L21-2 | Soil | KX344121 | 100 | 386/386 |
| 1166 | C7p3_ML_083 | Lake Pontchartrain | FJ353287 | 94 | 363/386 |
| 1168 | A1M11-otu2-11 | Groundwater from observation well | KY356865 | 96 | 372/386 |
| 1173 | mrR2.33 | Mackenzie River | DQ310444 | 98 | 376/385 |
| 1183 | A0610R002_A13 | Rice paddy soil | AB652183 | 90 | 349/386 |
| 1184 | Thp_A_134 | Thermopiles hot springs | EF444654 | 96 | 368/385 |
| 1185 | QYsA-41 | Brown soil | KP018490 | 99 | 382/386 |
| 1196 | B235106A02 | River water | FN864056 | 95 | 366/387 |
| 1219 | Wet11 | Wetland soil | KX856592 | 92 | 356/389 |
| 1223 | 21SuezARCH | Sediment | AB899917 | 89 | 348/390 |
| 1233 | 001 | DUSEL water from Homestake Mine | FJ718986 | 99 | 385/386 |
| 1239 | C7p3_ML_083 | Lake Pontchartrain | FJ353287 | 100 | 385/385 |
| 1267 | Sto-6_8-rtC12 | Storegga Slide sediment | GU055894 | 100 | 385/385 |
| 1270 | UT09 | Field soil | KC784843 | 99 | 383/385 |
| 1356 | Wet11 | Wetland soil | KX856592 | 95 | 362/383 |
| 1378 | MTG-45-162 | Cinnamon soil | JN900420 | 97 | 374/385 |
| 1410 | sagar171 | Indian continental shelf | JN030749 | 100 | 385/385 |
| 1416 | BE326FW120712BH2_arch_16S_rRNA_OTU8 | Subsurface borehole water | KF901397 | 95 | 365/384 |
| 1418 | Arc DX-Clone 35 | Dax-DX hot spring | FR727670 | 97 | 373/386 |
| 1419 | A113P0 | Paddy soil | KP327854 | 92 | 357/387 |
| 1422 | mrR1.55 | Mackenzie River | DQ310403 | 96 | 370/386 |
| 1438 | mrR1.15 | Mackenzie River | DQ310414 | 83 | 316/380 |
| 1450 | ANNA-C2 | Neuston of Lake Llebreta | FR820762 | 99 | 381/386 |
| 1464 | IG2C08 | Water | JF835217 | 93 | 359/384 |
| 1470 | 030-F12-JB 29745 | Biogas reactor | LT546394 | 100 | 386/386 |
| 1517 | A59P0 | Paddy soil | KP203026 | 98 | 377/385 |
| 1621 | Sv-30 | High arctic permafrost peat | AM712514 | 95 | 344/361 |
| 1623 | Arc16S_70 | Rice paddy soil | KY851270 | 100 | 386/386 |
| 1624 | mrR1.35 | Mackenzie River | DQ310385 | 98 | 377/386 |
| 1628 | W50mCGB726 | Serpentinized dunite | JN002482 | 98 | 379/385 |
| 1660 | LW-26 | Subglacial sediment | HQ214470 | 100 | 385/385 |
| 1675 | E114P700 | Paddy soil | KP328033 | 91 | 355/389 |
| 1681 | AC16Sarch47 | Arsenic contaminated sediment | HQ664150 | 100 | 386/386 |
| 1684 | B235006F07 | River water | FN864027 | 96 | 372/386 |
| 1685 | AR44-1_E08_F | Marine sediment | KT424915 | 87 | 335/385 |
| 1686 | Sto-0_6-rtF09 | Storegga Slide sediment | GU055866 | 92 | 357/386 |
| 1703 | MY-24-86 | Cinnamon soil | JN900404 | 96 | 371/386 |
| 1771 | B235106A02 | River water | FN864056 | 92 | 359/389 |
| 1817 | N8-A11 | Neuston of Lake Llebreta | FN691485 | 92 | 355/387 |
| 1875 | noRSD76T10_A46 | Rice paddy soil | KJ885410 | 98 | 375/384 |
| 1886 | "DGGE band HMLWC47" | Upper water column 1 m | AM749483 | 91 | 314/344 |
| *Thaumarchaeota* | | | | | |
| 851 | ATE1010 | Soil | KT461091 | 100 | 385/385 |
| 758 | SWA13 | Stream | AB294269 | 97 | 375/385 |
| 1000 | THAUM_IC_A072 | Soil | KF276295 | 100 | 385/385 |
| 460 | OTU254 | Wetland | KX077512 | 99 | 383/385 |
| 385 | UT38 | Field soil | KC784871 | 100 | 385/385 |
| 415 | ARCu-361 | Tibetan Plateau soil | GQ127528 | 100 | 385/385 |
| 323 | ARCu-530 | Tibetan Plateau soil | GQ127593 | 99 | 417/419 |
| 202 | Elev_16S_arch_998 | Rhizosphere soil | EF023105 | 100 | 385/385 |
| 829 | JSD6 | Arid desert sanddune | JQ071810 | 100 | 385/385 |
| 100 | Wet52 | Wetland soil | KX856631 | 100 | 385/385 |
| 104 | XXY_CC_AOA_5-2014 | Acidic soil | LC178528 | 100 | 385/385 |
| 1707 | GYsA-1 | Red soil | KP018451 | 99 | 357/359 |
| 29 | smkt_SCG_01_0002 | Ocean drilling core sample | AB801191 | 100 | 385/385 |
| 20 | ys_19 | Upland field soil | AB583888 | 100 | 385/385 |
| 18 | DGGE gel band A23 | Biofilm | KF041030 | 100 | 386/386 |
| 13 | Arch-Malhada4.1 | Pinus pinaster tree | KJ655204 | 100 | 386/386 |
| 39 | GJarc2_G06 | Soil of Gotjawal Forest | AB848904 | 100 | 385/385 |
| 16 | B148P50 | Paddy soil | KP327936 | 100 | 385/385 |
| 8 | THAUM_LC_B011 | Soil | KF275835 | 100 | 386/386 |
| 15 | THAUM_HG_B024 | Soil | KF276523 | 100 | 385/385 |
| 9 | THAUM_HG_B036 | Soil | KF276534 | 100 | 386/386 |
| 1354 | SLA-AM3-1 | Permafrost soil | JQ978502 | 99 | 383/385 |
| 267 | Elev_16S_arch_627 | Rhizosphere soil | EF022773 | 99 | 383/385 |
| 7 | OTU_337 | Anaerobic full-scale reactors | KU656235 | 100 | 385/385 |
| **1** | W5P2-D01 | Agricultural soil | GQ871406 | 100 | 386/386 |
| **2** | DT-14Q-2T-17 | Qilian Mountain | KR066470 | 100 | 385/385 |
| **3** | denovo2932 | Freshwater sediment | MF694278 | 100 | 386/386 |
| **4** | S2A-4 | Soil | KC753272 | 100 | 385/385 |
| **5** | B75 | Meadow soil | KX061161 | 100 | 385/385 |
| **6** | OTU226 | Soil | KX077485 | 100 | 385/385 |
| **1120** | L050020A08 | Air filter sample | KF683487 | 99 | 380/385 |

Note: the bold OTUs are the most abundant OTUs in the black soils.
