# Supplementary material for: Biogeographic Distribution Patterns of the Archaeal Communities Across the Black Soil Zone of Northeast China
Source: Front Microbiol. 2019 Jan 25;10:23. doi: 10.3389/fmicb.2019.00023 (PMC6355713; doi:10.3389/fmicb.2019.00023)
Supplement: Table S5 — The correlation coefficients (r) and significance levels (P) of pairwise 581 correlation between NMDS scores and soil total C content, and soil pH value. Values 582 in bold indicate significant correlations (P < 0.01). [file Table_5.DOC]

**Table S5 The correlation coefficients (*r*) and significance levels (*P*) of pairwise regressions between NMDS scores and soil total C content, and soil pH value. Values in bold indicate significant correlations (*P* < 0.01).**

| Soil parameter |  | Weighted NMDS | | | | |
| --- | --- | --- | --- | --- | --- | --- |
|  |  | NMDS1 | |  | NMDS2 | |
|  |  | *r* | *P* |  | *r* | *P* |
| pH |  | **-0.836** | <0.001 |  | -0.044 | 0.831 |
| Total C |  | -0.019 | 0.926 |  | **0.649** | <0.001 |
